# Using endometrial immune profiling in young IVF patients: implantation rates improved for morphologically sub-optimal embryos

**DOI:** 10.3389/fimmu.2026.1706032

**Published:** 2026-03-16

**Authors:** Nathalie Lédée, Nada J. Habeichi, Mona Rahmati, Geraldine Dray, Nicole Kerkhoven, Eric Vicaut, Abdourahmane Diallo, Nino Guy Cassuto, Lea Ruoso, Laura Prat-Ellenberg, Marie Petitbarat

**Affiliations:** 1 MatriceLab Innove Laboratory, Immeuble Les Gemeaux, Créteil, France; 2 Centre d’Assistance Médicale à la Procréation, Hôpital des Bluets, Paris, France; 3 Université Paris Saclay, UVSQ – UFR Simone Veil – Santé, Versailles, France; 4 London Women’s Clinic, London, United Kingdom; 5 Unité de Recherche Clinique, Hôpital Fernand Widal, (APHP), Université Paris-Diderot, Paris, France; 6 Laboratoire Drouot, Paris, France

**Keywords:** embryo quality, endometrial immune profiling, IVF, pregnancy, uterine immune regulation

## Abstract

**Introduction:**

A balanced endometrial immune profile is considered to be the ideal environment for a successful implantation, as opposed to endometrial immune dysregulation. However, the embryo quality may be the pivotal ‘immune’ factor compared to the uterine immune environment.

**Study method:**

After providing informed consent, young infertile patients involved in ART underwent an analysis of their endometrial immune profile before their embryo transfer (ET). Each ET was classified as a Top transfer if the used embryos were of Top morphology; all other transfers were classified as non-Top. The live birth rate (LBR) after ET was compared between patients with a balanced endometrial immune profile and patients with a dysregulated endometrial immune profile, with no intervention to regulate the endometrial environment (conventional care).

**Results:**

Overall, the LBR did not differ significantly between patients with balanced and dysregulated endometrial profile receiving conventional care (39.5% vs 29.7%; OR: 1.55 [0.66–2.72]). However, among patients with a non-Top transfer, the LBR was higher in the balanced group (38.4% vs 21.2%; OR: 2.11 [1.02–4.37]). In contrast, no difference was observed in the case of a Top transfer (46.3% vs 45.8%; OR: 0.99 [0.38–2,55]).

**Conclusion:**

These preliminary results suggest that a balanced endometrial immune environment may favour the implantation in patients using morphologically suboptimal embryos. In contrast, an endometrial immune dysregulation negatively impacts the implantation of morphologically suboptimal embryos.

**Trial registration:**

NCT02262117

**Trial registration date:**

October, 7th, 2014

**Date of the first patient’s enrolment:**

October, 30th, 2015

## 1 Introduction

Assisted reproductive technologies (ART) have made significant progress over the past few decades and are now widely recognized as an effective treatment for infertility. According to the World Health Organization (WHO), approximately 15% of couples engaging in unprotected intercourse, equivalent to 48 million couples and 186 million individuals worldwide, struggle with infertility (1). Despite these remarkable advances, ART success rates remain relatively low. The live birth rate (LBR) per initiated IVF cycle is approximately 30% for women under the age of 35, meaning a failure rate of approximately 70%, with success rates declining sharply as maternal age increases (2). This challenge contributes to considerable emotional, psychological, and financial stress, leading to substantial social and economic consequences, including reduced productivity and a diminished quality of life for affected couples (3).

Human implantation is a black box and involves the synchronized interaction of the embryo and the endometrium. The window of implantation (WOI) defines the crucial time frame of uterine receptivity when the endometrium undergoes changes in response to hormonal signals from the ovary, preparing it to receive and support an embryo (decidualization) (4). Uterine immune cells play a critical role in the process, as they contribute to the establishment of a receptive environment for the embryo to implant and develop (5, 6). During this WOI, a crucial shift from an adaptive immunity to an innate immunity takes place in the endometrium (7). This shift creates an immunologically tolerant and fruitful environment for the developing embryo, which is a semi-allograft (8). The balance between Th-1 and Th-2 cytokines plays an essential role in the success of implantation. The predominance of Th-2 cytokines over Th-1 cytokines favours the differentiation of local innate immune cells into cells that will enhance or impede implantation and placentation (9). Endometrial immune profiling is an innovative strategy involving the analysis of functional immune biomarkers in the endometrium. The quantification of RNA expression levels of five biomarkers gives key information regarding the immunoregulated Th-2/Th-1 local equilibrium, the remodelling of spiral arteries, and the mobilization and maturation of uterine natural killer (uNK) cells. Through the quantification of these targets, we define the endometrial immune profile during the mid-luteal phase. The objective is to understand if the endometrium is prepared for an effective implantation and to detect any endometrial immune imbalance which may impair the process of implantation (10).

In our recent randomised controlled trial (RCT), we revealed that applying precision care to rebalance the endometrial immune environment, can significantly increase LBR in good prognosis IVF patients diagnosed with endometrial immune dysregulation, compared to those with dysregulated endometrial immune profile who did not receive precision care (conventional standard care – control group) (11). Subclass analysis in this RCT revealed that mainly patients using embryos with suboptimal quality benefitted from precision care compared to conventional care (LBR 39.6% versus 21.3%; odds ratio (OR): 2.41 [1.28-4.61]). The same comparison for patients using top quality embryos showed a LBR of 42.61% versus 46.3% (OR 0.86 [1.05–3.43]). In the present study, we aim to compare, within the same cohort, the outcome of patients with a balanced immune environment to those with dysregulated endometrial immune profile who received conventional standard group, with no precision care applied.

In previous large cohort studies, including patients with a history of recurrent implantation failure, we observed that patients with a balanced immune environment had a lower LBR when compared to those with dysregulated endometrial immune profile receiving precision care (12–14). Our interpretation was that in patients with a balanced immune environment and a history of recurrent failures, the cause of failure was directly related to the poor quality of the embryos, which compromised consequently pregnancy. In contrast, in good prognosis IVF cohort, we anticipate a higher LBR in patients with a balanced immune profile, as there is no underlying factor for generating embryos with a limited outcome (age below 38 years, normal ovarian reserve).

More importantly, there is a growing tendency in the field of IVF to focus increasingly on “embryo quality.” This trend leads to more invasive, selective, and costly testing of blastocysts, combined with a “keep calm and carry on trying” approach, while undermining the role of the endometrium in implantation success (15). The impact of an adapted endometrium to establish a favourable cross talk for a successful implantation, even in presence of “sub-optimal” embryos (i.e. limited morphology and genetically untested) definitely needs to be investigated.

## 2 Methods

### 2.1 Study design

The study is designed as a prospective cohort study including young patients, from infertile couples, with a normal ovarian reserve, undergoing ART, with documented assessment of their uterine immune environment prior to a scheduled ET.

In our previous published RCT, patients diagnosed with a dysregulated endometrium were randomised: half of the patients received conventional medical care (regardless of their endometrial immune profile) while the other half received a personalized medical care (taking in account their endometrial immune profile documented during the initial check-up).

In the current study, the outcome of patients with a balanced uterine immune profile was compared to those with a dysregulated uterine immune profile who underwent conventional standard medical care, meaning that the endometrial immune profile was not taken into consideration.

The study protocol was approved by Institutional Review Boards at the University Paris Diderot (clinicaltrials.gov NCT02262117) and our trial followed the extended CONSORT guidelines.

As previously described in detail (11), the first patient was included on October 30, 2015, and the last patient was included on February 8, 2023.

A Data and Safety Monitoring Board (DSMB) reviewed interim results periodically throughout the study. No change was made to the personal treatment design during all the whole study period.

All patients were followed at the same ART Unit (Hospital Pierre Rouques Les Bluets, Paris, France) for the endometrial biopsy, the oocyte retrieval and the embryo transfer.

The protocol was interrupted during the COVID pandemic in 2020.

#### 2.1.1 Time-0: consent- inclusion criteria and information

##### 2.1.1.1 Inclusion criteria

Eligible participants were infertile patients indicated for *in vitro* fertilization (IVF) with or without intracytoplasmic sperm injection (ICSI). The indications for IVF included tubal factor infertility, endometriosis, ovarian dysfunction, or unexplained infertility with failed intrauterine insemination (IUI). Of note, ICSI was indicated in cases of male infertility (oligo-astheno-teratospermia) or previous failure of oocyte fertilization in conventional IVF.

Patients had to be ≤38 years old at the time of inclusion and had a normal ovarian reserve, defined as anti-Müllerian hormone (AMH) >1.5 ng/mL, follicle-stimulating hormone (FSH) <10 IU/L on day 3, and an antral follicle count (AFC) >6 on day 3 of the menstrual cycle, as assessed by ultrasound. The number of prior oocyte retrievals for IVF/ICSI had to be strictly fewer than three. If a previous live birth had occurred in the past by IVF, the range of oocyte retrieval for the new attempt was 1. All patients had signed an informed consent form and had medical insurance.

##### 2.1.1.2 Exclusion criteria

Exclusion criteria were azoospermia or cryptozoospermia in the patient’s partner, uterine malformations, IVF/ICSI treatment planned at another ART facility, contraindications to corticosteroids, human chorionic gonadotropin (hCG), or slow intralipid infusions.

##### 2.1.1.3 Study procedure

If the inclusion criteria have been met and exclusion criteria were absent, the clinician informed the patient of the proposed protocol. If patients agreed to participate, an endometrial biopsy was scheduled in the mild luteal phase.

#### 2.1.2 Time 2: endometrial biopsy: collection and analysis

Endometrial biopsies were collected during the mid-luteal phase. Tissue samples were gently aspirated using a Pipelle de Cornier, which was rotated within the endometrial cavity (Leclair et al., 2011). The collected sample was then divided into two parts: The first portion was fixed in 4% buffered formaldehyde (QPath Formol 4%, VWR Chemicals, Fontenay-sous-Bois, France) for endometrial dating (Acosta et al., 2000) and CD56 immunolabeling. The second portion was preserved in RNAlater stabilization solution (MatriceLab Innove, France) for subsequent immunological analysis. Importantly, all samples were shipped at room temperature through postal services.

##### 2.1.2.1 RNA extraction and reverse transcription

Following confirmation of the histological dating, RNA was extracted from the biopsy sample conserved in RNAlater (Qiagen). RNA extraction was performed using the Biomek1.5 system with the Kit RNAdvance Tissue (Beckman-Coulter*).* The RNA was reverse-transcribed into cDNA with the First Strand cDNA Synthesis Kit for RT-PCR (Roche, Meylan, France), according to the manufacturer’s instructions. The cDNAs were stored at –20 °C until use.

##### 2.1.2.2 Quantitative RT-PCR

Quantitative RT-PCR was performed with a Light Cycler 480 instrument (Roche Diagnostic) and the Light Cycler 480 SYBR Green I Master mix (Roche Diagnostic). Final concentrations for reaction set-up were 0.5 µM of forward and reverse primers and 1/20 of diluted cDNA. Cycling conditions were as follows: denaturation (95 °C for 5 min), amplification and quantitation (95 °C for 10 s, 60 °C for 10 s and 72 °C for 15 s) repeated 40 times, a melting curve program (65-95 °C with a ramp rate of 2.2 °C/s) and a cooling step to 4 °C. Each quantitative RT-PCR assay included a negative control without cDNA and inter-run calibrator (IRC) samples as a positive control. The IRC for all the primers (Interleukin-18, Interleukin-15, TNF- weak-inducer of apoptosis; Fn14 and CD56) was obtained from pools of RNA endometrial samples. The IRC cDNA, after dilution by a factor of 20, underwent the same quantitative RT-PCR protocol as the unknown samples. PCR efficiency for each quantified target and reference was calculated with known serial dilutions of each specific cDNA. LightCycler^®^480 Software release 1.5.0 was used to analyse data, and each specific target transcription level was normalized to the geometric mean of the transcription level of the reference gene RPL13A and B2M, with the software’s advanced relative quantification workflow. Gene amplification efficiency was specifically determined. For each sample, the results were expressed as the ratio of target/reference cDNA.

##### 2.1.2.3 Immunohistochemistry of uNK cells

IHC was carried out on 5-µm thick slides of biopsy sample tissue preserved in 4% formalin, using an automated streptavidin-biotin method (Benchmark GX, Ventana Medical Systems).The prediluted anti-CD56 (clone 123C3) murine monoclonal primary antibody (Ventana Medical Systems^®^, Roche Diagnostics) was applied according to the manufacturer’s instructions. Briefly, after deparaffinization of the slides, antigen retrieval was performed for 60 min in a pH 8.4 Cell Conditioning 1 solution. The slides were then incubated in CD56 primary antibody for 32 min. Slides for negative controls were prepared by replacing the primary antiserum with non-immune IgG. Slides were then incubated for 8 min with a biotinylated antimouse secondary antibody. Diaminobenzidine or 3-amino-9-ethylcarbazole was used as the chromogen (iVIEW DAB detection kit, Ventana Medical Systems) and slides were counterstained with hematoxylin for 2 min, incubated in bluing reagent (for 2 min), and finally mounted. Of note, after each step, the slides were rinsed with a reaction buffer. The uNK cell count was measured as the mean of CD56^+^ cells in 4 representative fields at ×400 magnification.

To establish the endometrial immune profile, a step‐by‐step procedure was followed beginning with the IL‐18/TWEAK mRNA ratio (indicating local angiogenesis and possibly a Th1 deviation), then the CD56^+^ cell count (reflecting uNK cell mobilization), and finally the IL‐15/Fn‐14 mRNA ratio (indicative of uNK cell maturation and uNK cytotoxic activation).

#### 2.1.3 Time-3: interpretation of analysis and constitution of our studied group

The patent untitled “METHOD FOR INCREASING IMPLANTATION SUCCESS IN ASSISTED FERTILIZATION” [PCT/EP2013/065355] described the method for determining a uterine immune profile and defined the norms of expression for our biomarkers in a fertile cohort, and documented that an immune profile was reproducible from one cycle to another over a period of six months if no surgery or pregnancy occurred in the meantime.

Endometrial immune profiles can be classified into four types:

A balanced endometrial immune profile, characterized by IL-18/TWEAK and IL-15/Fn-14 mRNA ratios and a CD56+ cell count within the same range as previously defined in the fertile cohort. This profile suggests that the endometrium is ready to go through the following steps of implantation, including apposition, adhesion, and invasion. The three other subgroups represent the dysregulated patients were randomised through the electronic server (Cleanweb- APHP)

All the others profile are considered as dysregulated

An under-activated endometrial immune profile was characterized by at least one of the three biomarkers (IL 18/TWEAK ratio, CD56 expression level, and/or IL-15/Fn14 ratio) being low and the other two biomarkers either normal or low. This profile suggests that the endometrium is not fully effective for adhesion and promoting adequate immunotrophism during initial placentation.

An over-activated endometrial immune profile was characterized by at least one of the three biomarkers (IL 18/TWEAK ratio, CD56 expression level, and/or IL-15/Fn14 ratio) being high, with IL 18/TWEAK ratio and IL-15/Fn14 ratio being either normal or high.

The mixed endometrial immune profile is a subtype of over-immune activation. The mixed profile was distinguished by a high IL-18/TWEAK ratio (excess Th-1 cytokines) and a low IL-15/Fn14 mRNA ratio (reflecting immature NK cells).

The over-activated and mixed profiles suggest that the endometrium is not prepared for the crucial step of trophoblast invasion and may be in a state that can reject the embryo because of a cytotoxic activation of uNK cells in LAKs (lymphocyte-activated killer cells).

Only patients with endometrial immune dysregulation have been randomised. half of them were assigned for conventional-standard care. This group was then compared to patients with a balanced immune profile.

The patient will have a standard embryo transfer without scratching, adjunction of corticoids/intralipids/gonadotropins chorionic or double transfer.

#### 2.1.4 Time 5: preparation for the embryo transfer

IVF/ICSI after ovarian monitored hyper-stimulation for a fresh embryo transfer as well as endometrial preparation for freeze embryos were conducted classically.

The delay between the last immunologic analysis and the embryo transfer had not exceed 9 months. Patients were excluded from the analysis. if a spontaneous pregnancy or a gynaecological surgery occurred between the biopsy and ET.

##### 2.1.4.1 Embryo transfer policy applied

Before 2018, Embryo culture until day-5 was not routinely applied as a first-line policy of transfer; day-2/3 embryos at the cleavage stage were typically transferred. If no transfer has been previously done, one day-3 embryo was transferred below 30 years but in majority two day-2/3 embryos were transferred.

After 2018, the embryo transfer policy has been to favour prolonged culture of embryos until day-5 to promote singletons and prevent multiple pregnancies. Day-3 transfers were only performed if less than two embryos were available on day 3 or in case of previous prolonged culture failure.

In patients with a balanced immune profile or a dysregulated immune profile in conventional care, uterine immune profiling did not impact the classical embryo transfer policy. Embryologists were blind regarding the precise diagnosis of uterine dysregulation.

##### 2.1.4.2 Impact of embryo quality

To evaluate the impact of embryo quality on subsequent pregnancy rates, embryo transfers (ETs) were organised into two classes (top transfer or non-top transfer) based on the embryologist’s observations on the day of transfer (16, 17). The same embryologists (LR and GC) were responsible for selecting the embryos for transfer or freezing throughout the study. An external quality evaluation was conducted twice a year to ensure that no deviations were observable in the classification of embryo quality according to the international classifications.

On day 2-3, the standard BLEFCO classification (16) was used to evaluate the embryos and on day 5 the Gardner classification (17) was used for the evaluation of blastocyst quality. On day-2/3, a Top-grade high-quality embryo was defined as an embryo with typical equal-sized blastomeres (cells) or unequal-sized blastomeres (cells) according to the number of cells with less than 10% of fragmentation. On day-2 the embryo should have 2 to 4 cells, and 6 to 10 on day-3. On day-5, Top grade excellent quality blastocyst was defined as a blastocyst with large, fully expanded blastocoel (fluid-filled cavity), and inner cell mass (ICM) and trophectoderm (TE) tightly packed and clearly defined [B5AA-B5AB-B5BA-B4AA-B4AB-B4BA].

Top transfers were defined as the transfer of Top-quality embryos and no-top transfer as the transfer of non-top embryos. If two embryos were transferred, both embryos had to be evaluated as Top quality to be a top transfer. If one embryo was top and the other non-top, the transfer was classified as non-top.

#### 2.1.5 Time 6: outcome analysis of the embryo transfer attempt

LBR was defined by the birth of a living baby and was the primary outcome of the mITT analysis. Secondary outcomes were the ongoing pregnancy rate, the clinical pregnancy rate and the miscarriage rate

The ongoing pregnancy rate was defined by an ultrasonic evaluation attesting of the presence of a yolk sac with cardiac activity at 12 weeks of amenorrhea that has progressed beyond the first trimester (12 weeks) and was still continuing. The clinical pregnancy rate was defined by a βhCG over 100 IU/l in the serum 10 to 12 days after the embryo transfer. Miscarriage referred to the loss of a pregnancy that occurred after the transfer of an embryo(s) or the attempt at any stage of pregnancy, from implantation to the end of the first trimester (12 weeks gestation). Miscarriage did not include biochemical pregnancies that were considered as no pregnancy in our analysis but included early pregnancy losses (gestational sac seen on ultrasound but no heartbeat).

### 2.2 Statistical methodology

In this prespecified subgroup analysis, we assessed the effect of types of endometrial immune profile on LBR according to the quality of embryo transfer. Categorical data are presented as numbers (percentages). Continuous variables are presented as means with standard deviations. Baseline characteristics are shown in patients with Top and non-Top quality of ET at inclusion and between endometrial immune profile groups. Categorical baseline characteristics were compared using Pearson’s chi-square test or Fisher’s exact test if appropriate. Continuous baseline characteristics were compared using Welch–Satterthwaite test.

The primary outcome LRB was compared between the endometrial immune profile groups using Firth’s penalized likelihood logistic regression model with fixed effects for endometrial immune profile types, quality of ET and a endometrial immune profile types -by-quality of ET interaction term. Odds ratios, associated *P* values for odds ratios, 95% CIs, and the *P* values for interaction were presented. Models were run with and without adjustment for baseline covariates prespecified as potential confounders. The adjusted model included age, BMI, endometrial thickness, AMH, type of ET, stage and number of embryos at the time of ET, and stimulation protocol. Additional, prespecified exploratory subgroup analyses were conducted by age and by previous failed ET. These analyses were performed using Firth’s penalized likelihood logistic regression without adjustment for baseline covariates. Analyses have not been adjusted for multiple comparisons due to several exploratory subgroup analyses and should not be used to infer definitive treatment effects.

All hypothesis tests were two-sided, and *P* value <.05 was interpreted as statistically significant. All tests will be performed using SAS version 9.4.

## 3 Results

### 3.1 Flow-chart of the patients included

**Figure 1:**
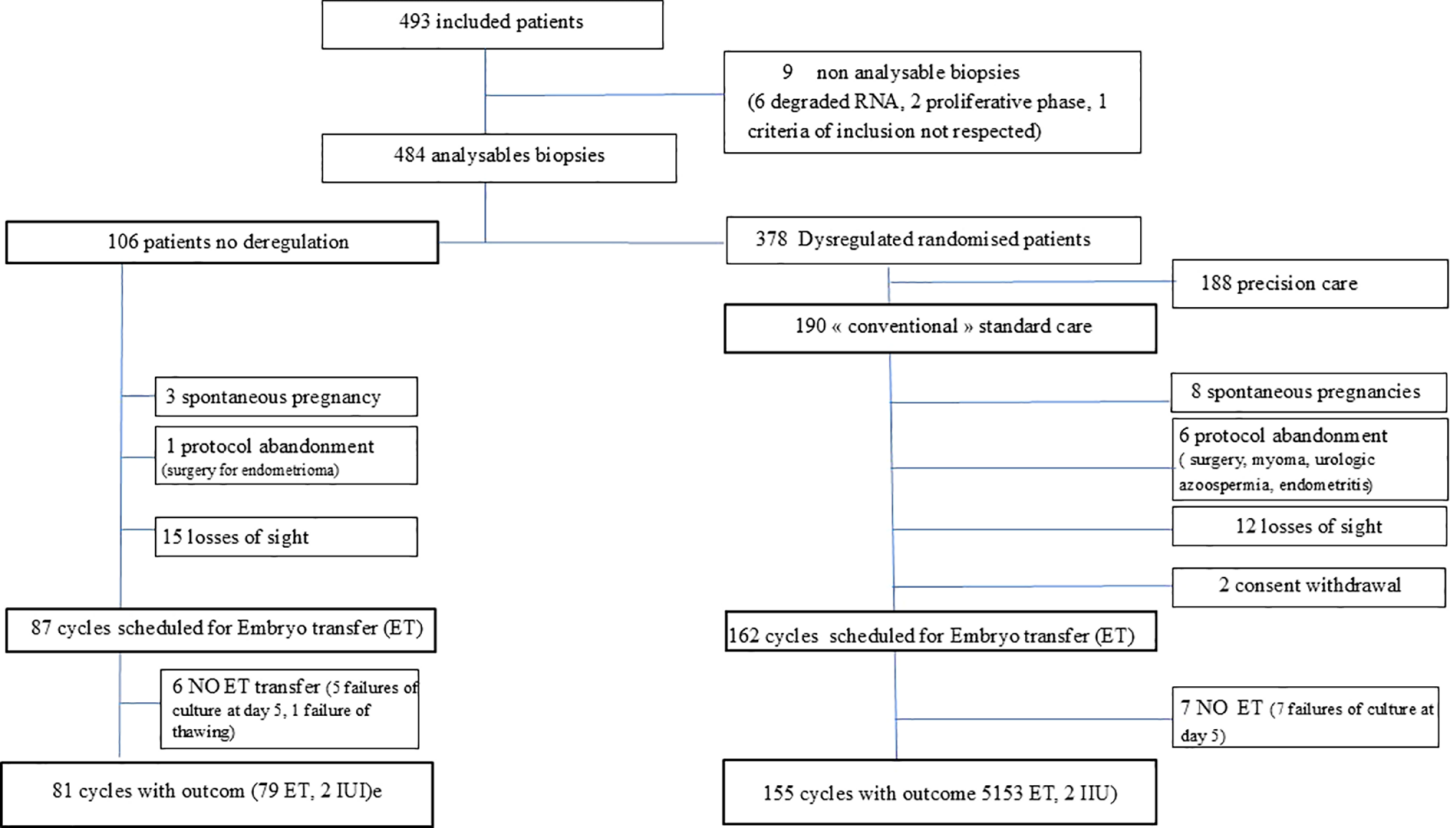
Flow chart of the study.

**Figure 1** illustrates the progress of patients through the trial. 493 patients were included in this study from October 30^th^, 2015 to February 8^th^, 2023. The immune profiling analysis, however, was successfully performed for only 484 patients.

Out of the 484 patients, 22% (106) had a balanced endometrial immune profile, and 78% (378) had a dysregulated endometrial immune profile. Among the dysregulated patients, 190 were randomised to receive conventional standard treatment.

In the balanced group, 25 patients were not included in the ITT study. 3 were spontaneously pregnant, 1 had a surgery for endometriosis, 15 were loss of sight and 5 did not had a blastocyst after embryo culture and 1 embryo failed at the thawing step. In the dysregulated group with conventional care, 35 patients were not included in the ITT analysis. 8 were spontaneously pregnant, 6 had a surgery before ET, 12 were loss of sight, 2 withdraw their consent, and 7 failed to have a blastocyst after the embryo culture.

Outcomes were available for 236 patients: 81 balanced and 155 dysregulated, all receiving a conventional standard care.

### 3.2 Demographic characteristics, aetiology and medical history of patients

**Table 1** summarizes the clinical and demographic characteristics, as well as embryo transfer parameters, of balanced and dysregulated patients randomized to standard conventional care. No significant differences were observed between the two groups.

**Table 1:** Patients characteritics at baseline according to the immune profile.

Patients characteristics	Total N=236	Balanced N=81	Dysregulated N=155	P value	
Age, years	33.2 ± 3.3	33.2 ± 3.4	33.2 ± 3.2	0.954	^25^
Age, class — no. (%)				0.462	^13^
≥ 35	80 (33.9%)	30 (37.0%)	50 (32.3%)		
< 35	156 (66.1%)	51 (63.0%)	105 (67.7%)		
BMI, kg/m²	23.9 ± 4.5	23.8 ± 4.8	23.9 ± 4.4	0.898	^25^
AMH, ng/ml	4.25 ± 4.03	4.02 ± 2.74	4.37 ± 4.58	0.469	^25^
Number of previous oocytes pick-up	0.8 ± 0.7	0.7 ± 0.6	0.8 ± 0.7	0.194	^25^
Number of previous embryos transferred	1.2 ± 1.2	1.1 ± 1.3	1.3 ± 1.2	0.391	^25^
Previous ET failure, two levels — no. (%)				0.321	^13^
No previous ET	86 (36.4%)	33 (40.7%)	53 (34.2%)		
At least 1 ET failure	150 (63.6%)	48 (59.3%)	102 (65.8%)		
Previous ET failure, three levels — no. (%)				0.463	^13^
Two or more transfer failed	91 (38.6%)	27 (33.3%)	64 (41.3%)		
One transfer failed	59 (25.0%)	21 (25.9%)	38 (24.5%)		
No previous ET	86 (36.4%)	33 (40.7%)	53 (34.2%)		
Stimulation protocol — no. (%)				0.326	^15^
Antagonist	142 (60.2%)	46 (56.8%)	96 (61.9%)		
Long Agonist	37 (15.7%)	14 (17.3%)	23 (14.8%)		
Spontaneous cycle	31 (13.1%)	15 (18.5%)	16 (10.3%)		
Substituted cycle	22 (9.3%)	5 (6.2%)	17 (11.0%)		
FSH	4 (1.7%)	1 (1.2%)	3 (1.9%)		
Endometrial thickness at transfer, mm	9.8 ± 2.2	9.8 ± 2.0	9.8 ± 2.4	0.809	^25^
Overall quality of the embryo transfer — no./total no. (%)				0.453	^13^
Top	78/232(33.6%)	24/79(30.4%)	54/153(35.3%)		
No Top	154/232(66.4%)	55/79(69.6%)	99/153(64.7%)		
Type of Embryo transfer — no./total no. (%)				0.459	^13^
Fresh	177/232(76.3%)	58/79(73.4%)	119/153(77.8%)		
Freezed-thawed	55/232(23.7%)	21/79(26.6%)	34/153(22.2%)		
Stage and number of embryos at the time of ET — no./total no. (%)				0.192	^15^
1 Day 2/3 ET	4/232(1.7%)	1/79(1.3%)	3/153(2.0%)		
2 Day-2/3 ET	49/232(21.1%)	19/79(24.1%)	30/153(19.6%)		
1 Day 5–6 ET	165/232(71.1%)	51/79(64.6%)	114/153(74.5%)		
2 Day 5–6 ET	11/232(4.7%)	7/79(8.9%)	4/153(2.6%)		
Double sequential embryo transfer	3/232(1.3%)	1/79(1.3%)	2/153(1.3%)		

^25^WelchSatterthwaite test.

^15^Fisher’s exact test.

^13^Pearson’s chi-square test.

The mean age of the cohort was 33 years old. 66% were below 35 years old and 33.8% over 35 years old.

The main causes of infertility in the cohort were as follow: 38.5% male infertility, 20%, tubal related pathology, 14% ovulatory problem, 12% idiopathic, 9% endometriosis, and 1.5% recurrent miscarriages. Of note, 5% of the infertility aetiology was mixed male and female factors.

For the 236 patients with available outcomes, 36.4% (86/236) never had an ET before, 25% (59/236) previously failed to achieve pregnancy after one ET, 26% (62/236) after two ET and 12% (29/236) after more than two (3-5).

According to the new definition of repeated implantation failures (RIF) edited by the ESHRE committee (18) in 2023, 4.6% (11/236) of the patients included could be classified as RIF patients in this cohort.

### 3.3 Quality and number of embryos transferred

A total of 153 embryo transfers in the conventional group (64% non-Top) and 79 were performed in the balanced group (69% non-Top).

**Table 2** summarizes the clinical and demographic and embryonic data for Top versus non-top transfers. Significant differences were observed between Top and Non-top transfers regarding the protocol, type of transfer (fresh or frozen) and stage and number of embryos transferred. The analysis included an adjusted logistic regression that considers these factors. In the conventional group, 75.8% of embryos were at the blastocyst stage compared to 73.7% in the balanced group. This population will be used for the sensitivity analysis, which will adjust for the calendar year of the blastocyst transfer.

**Table 2:** Patient characteristics at baseline according to the quality of the embryo transfer.

Patients characteristics	Total N=232	Top transfers N=78	No top transfers N=154	P value	
Age, years	33.2 ± 3.3	32.5 ± 3.3	33.6 ± 3.2	0.023	^25^
Age, class — no. (%)				0.034	^13^
≥ 35	78 (33.6%)	19 (24.4%)	59 (38.3%)		
< 35	154 (66.4%)	59 (75.6%)	95 (61.7%)		
BMI, kg/m²	23.9 ± 4.5	24.3 ± 4.9	23.6 ± 4.3	0.265	^25^
AMH, ng/ml	4.26 ± 4.06	3.90 ± 2.45	4.44 ± 4.66	0.254	^25^
Number of previous oocytes pick-up	0.8 ± 0.7	0.7 ± 0.7	0.8 ± 0.6	0.706	^25^
Number of previous embryos transferred	1.2 ± 1.2	1.2 ± 1.4	1.2 ± 1.2	0.871	^25^
Previous ET failure, two levels — no. (%)				0.324	^13^
No previous ET	85 (36.6%)	32 (41.0%)	53 (34.4%)		
At least 1 ET failure	147 (63.4%)	46 (59.0%)	101 (65.6%)		
Previous ET failure, three levels — no. (%)				0.366	^13^
Two or more transfer failed	90 (38.8%)	31 (39.7%)	59 (38.3%)		
One transfer failed	57 (24.6%)	15 (19.2%)	42 (27.3%)		
No previous ET	85 (36.6%)	32 (41.0%)	53 (34.4%)		
Stimulation protocol — no. (%)				0.015	^15^
Antagonist	138 (59.5%)	56 (71.8%)	82 (53.2%)		
Long Agonist	37 (15.9%)	12 (15.4%)	25 (16.2%)		
Spontaneous cycle	31 (13.4%)	8 (10.3%)	23 (14.9%)		
Substituted cycle	22 (9.5%)	2 (2.6%)	20 (13.0%)		
FSH	4 (1.7%)	0 (0.0%)	4 (2.6%)		
Endometrial thickness at transfer, mm	9.8 ± 2.2	9.8 ± 2.1	9.8 ± 2.3	0.856	^25^
Type of Embryo transfer — no./total no. (%)				0.006	^13^
Fresh	177/232 (76.3%)	68/78 (87.2%)	109/154 (70.8%)		
Freezed-thawed	55/232 (23.7%)	10/78 (12.8%)	45/154 (29.2%)		
Stage and number of embryos at the time of ET — no./total no. (%)				0.010	^15^
1 Day 2/3 ET	4/232 (1.7%)	2/78 (2.6%)	2/154 (1.3%)		
2 Day-2/3 ET	49/232 (21.1%)	12/78 (15.4%)	37/154 (24.0%)		
1 Day 5–6 ET	165/232 (71.1%)	64/78 (82.1%)	101/154 (65.6%)		
2 Day 5–6 ET	11/232 (4.7%)	0/78 (0.0%)	11/154 (7.1%)		
Double sequential ET	3/232 (1.3%)	0/78 (0.0%)	3/154 (1.9%)		

^25^Welch–Satterthwaite test.

^15^Fisher’s exact test.

^13^Pearson’s chi-square test.

Data are mean ± SD, n(%). Where not all patients had available data, data are shown as n/N(%), mean ± SD [number of patients with available data].

### 3.4 Uterine immune profiles among balanced and dysregulated participants randomised in conventional standard care

106 patients had a balanced endometrial immune profile, and 190 patients had a dysregulated endometrial immune profile. Both groups were assigned to conventional standard care.

No significant differences were observed between the balanced and dysregulated conventional groups regarding age, BMI, AMH, previous ETs, endometrial thickness, fresh or frozen-thawed transfers, protocols used, stage and number of ET, transfer quality (**Table 1**).

Among the 155 dysregulated patients randomised for conventional standard care, 42% (65/155) had under-activate profiles, 48% (74/155) had over-activate profiles, and 10% (16/155) had mixed profiles.

### 3.5 LBR following conventional standard care, in balanced versus dysregulated groups, with TOP and non-TOP transfers

The impact of endometrial immune dysregulation on subsequent LBR was evaluated by comparing outcomes between patients with a balanced immune profile (n = 81) and those with dysregulated profiles (n = 155), all receiving conventional standard care.

When all patients were considered, no significant increase in LBR was observed in the balanced group compared with dysregulated patients who received conventional standard care (39.5% vs. 29.7%; OR: 1.55 [0.66–2.72]).

However, outcomes differed when we studied the effect of balanced versus dysregulated uterine environment on LBR according to quality of transfer (top or non-top) (**Table 3**). Following non-Top embryo transfer, unadjusted analysis revealed a significantly higher live birth rate (LBR) in the balanced group compared with dysregulated patients (38.4% [20/55] vs. 21.2% [21/99]; OR 2.11, 95% CI 1.02–4.37).

**Table 3:** Unadjusted logistic regression of the effect of balanced versus dysregulated status on live birth rate according to embryo transfer quality.

Immune profile	Balanced	Dysregulated	Unadjusted logistic regression model^†^		
			OR (95% CI^‡^)	P value	P value*
Live birth rate					0.218
No Top	20/55 (36.4%)	21/99 (21.2%)	2.11 (1.02 to 4.37)	0.044	
Top	11/24 (45.8%)	25/54 (46.3%)	0.99 (0.38 to 2.55)	0.976	

Values are no.(%). CI denotes confidence interval. ET denotes embryo transfer. OR denotes odds ratio.

^*^ The P value is from Firth’s penalized likelihood approach for testing the interaction between the treatment group and quality of ET.

^†^ Shown is a odds ratio calculated using a Firth’s penalized likelihood approach.

^‡^ CIs presented were profile likelihood-based confidence intervals.

As detailed in **Table 4**, after adjustment for age, BMI, endometrial thickness, AMH, and type of transfer, the odds ratio remained significant (OR 2.11, 95% CI 1.02–4.41). However, when protocols as well as the number and stage of embryo transfer were included in the model, the association was no longer statistically significant (OR 1.82, 95% CI 0.86–3.88).

**Table 4:** Adjusted logistic regression of the effect of balanced versus dysregulated status on live birth rate according to embryo transfer quality.

Immune profile	Balanced	Dysregulated	Adjusted logistic regression model^†^			Adjusted logistic regression model^††^		
			OR (95% CI^‡^)	P value	P value*	OR (95% CI^‡^)	P value	P value*
Live Birth rate					0.209			0.319
No Top	20/55 (36.4%)	21/99 (21.2%)	2.11 (1.02 to 4.41)	0.046		1.82 (0.86 to 3.88)	0.118	
Top	11/24 (45.8%)	25/54 (46.3%)	0.93 (0.34 to 2.51)	0.890		0.93 (0.33 to 2.59)	0.898	

Values are no.(%). CI denotes confidence interval. BMI denotes body-mass index. ET denotes embryo transfer. AMH denotes anti-Müllerian hormone. OR denotes odds ratio.

^*^The P value is from Firth’s penalized likelihood approach for testing the interaction between the treatment group and quality of ET.

^†^Shown is a odds ratio calculated using a Firth’s penalized likelihood approach with adjustment for age, BMI, Endometrial thickness, AMH, and type of ET.

^††^Shown is a odds ratio calculated using a Firth’s penalized likelihood approach with adjustment for age, BMI, Endometrial thickness, AMH, type of ET, stage and number of embryos at the time of ET, and stimulation protocol.

^‡^CIs presented were profile likelihood-based confidence intervals.

To test the hypothesis that unknown uterine dysregulation has a deleterious effect when morphologically suboptimal embryos are transferred, sensitivity analyses were performed on data restricted to blastocyst transfers (**Table 5**). Transfers of cleavage embryos, as well as double sequential embryo transfers, were excluded. The models were adjusted for the calendar year of blastocyst transfer to account for the eight-year study period and enhance the robustness of the findings. For patients with a non-top blastocyst transfer, the live birth rate (LBR) was significantly lower in the dysregulated group than in the balanced group (20.5% versus 38.5%, odds ratio [OR]: 2.63 [1.08-6.55]). In contrast, no difference was observed for TOP blastocyst transfers, whether dysregulation was present or absent (57.9% versus 51.1%, OR: 0.95 [0.32 to 2.93]).

**Table 5:** Sensitivity analysis restricted to blastocyst on the effect of balanced versus dysregulated uterine environment on LBR according to quality of ET.

Immune profile	Balanced	Dysregulated	Adjusted logistic regression model^†^		
			OR (95% CI^‡^)	P Value	P Value*
Live Birth rate					0.173
No Top	15/39 (38.5%)	15/73 (20.5%)	2.63 (1.08 to 6.55)	0.035	
Top	11/19 (57.9%)	23/45 (51.1%)	0.95 (0.32 to 2.93)	0.933	

Values are no.(%). CI denotes confidence interval. ET denotes embryo transfer. OR denotes odds ratio.

^*^The P value is from Firth’s penalized likelihood approach for testing the interaction between the treatment group and quality of ET.

^†^Shown is a odds ratio calculated using a Firth’s penalized likelihood approach with adjustment for calendar year of blastocyst transfer.

^‡^CIs presented were profile likelihood-based confidence intervals.

For Top blastocyst transfer, no difference in LBR was noted between the two groups (46.3% [11/24] vs. 45.8% [25/54]; OR: 0.98 [0.37–4.61]). Of note, as the number of Top embryos in a balanced environment is limited, this data should be considered with caution.

### 3.6 Comparison of forest plot analysis in balanced vs dysregulated patients receiving conventional care, and dysregulated patients receiving conventional vs precision care

**Figure 2:**
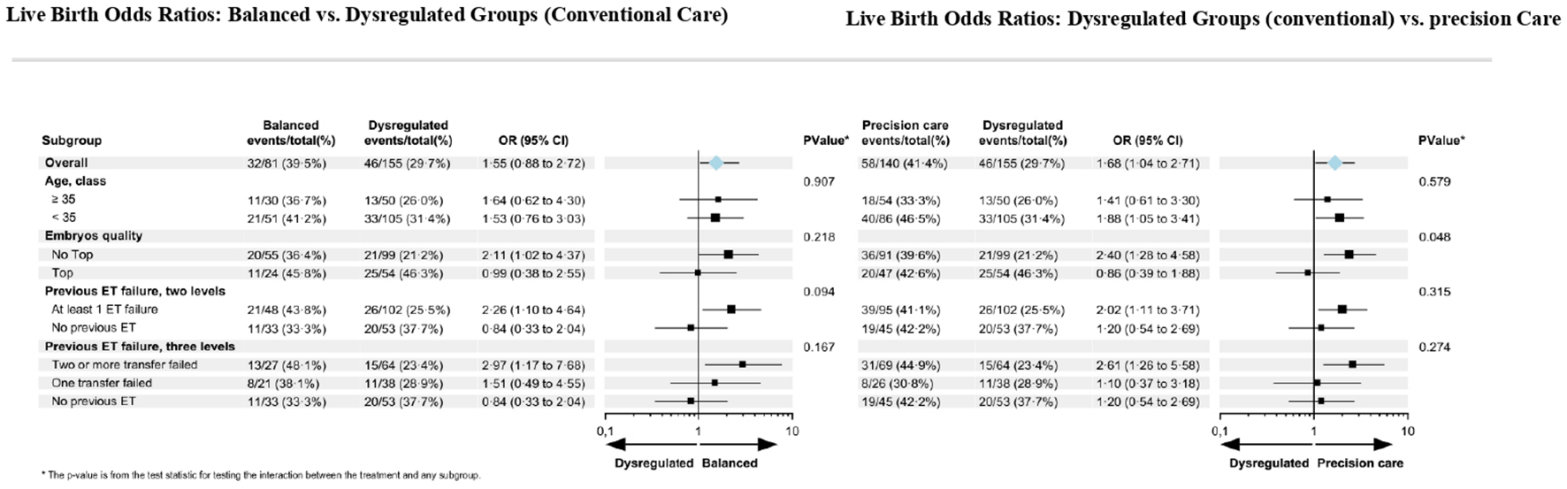
Forest plot of subgroup analyses according to patient characteristics and endometrial profile.

Previously, we published the forest plot analysis of dysregulated patients receiving precision care versus conventional care (11). Here, we juxtaposed the forest plot analyses of balanced versus dysregulated patients receiving conventional care, and of dysregulated patients receiving conventional versus precision care, to explore the differences and similarities between the groups. (**Figure 2**).

Following conventional care, when considering balanced vs dysregulated groups, we observed the same pattern of variations for non-Top transfers and for the ones with a history of ET failure. This was previously reported between the dysregulated groups receiving precision care vs conventional care.

When considering patients who failed to implant at least after one ET, a marked difference in LBR was observed. The LBR in balanced patients was 43.8% vs. 25.5% in dysregulated patients (OR: 2.27 [1.10-4.69]. The difference between the two groups studied became significantly higher when two previous ETs failed (LBR 48% vs. 23%, OR:3.03 [1.17-7.85]).

## 4 Discussion

The effect of endometrial immune dysregulation (without precise care to restore local balance) on LBR has never been documented. In previous large cohort studies, which mainly included patients with a history of repeated implantation failure or recurrent miscarriages, we were unable to collect detailed information on the stage, type (fresh or frozen) and quality of the embryos transferred. To the best of our knowledge, this is the first clinical trial assessing the role of a balanced endometrial immune profile on patients receiving conventional standard care.

Based on our findings, the LBR was not significantly increased in the overall population included. However, the power of the analysis was weakened by the reduced number of patients with a balanced immune environment (representing only 22% of the patients included). In this IVF cohort, the subgroup analysis suggested that a balanced endometrial immune environment could be a key factor improving LBR when non-Top embryos were transferred, as well as when patients had a history of one failed implantation. These findings should be regarded as hypothesis-generating, as statistical significance was lost after adjustment for stimulation protocol, embryo stage, and number of embryos transferred. Nevertheless, sensitivity analyses restricted to blastocyst transfers and adjusted for calendar year supported the same hypothesis. A multicentre, pair-matched controlled study (NCT06503952) is currently underway, enrolling exclusively patients undergoing single blastocyst transfer with stringent matching criteria derived from the present study to test this hypothesis in a real-world setting. The primary objective is to robustly evaluate the effect of precision-based care tailored to specific endometrial dysregulation profiles and to identify which embryo characteristics require a balanced uterine immune environment for successful implantation.

We observed identical prognostic factors (as the number of attempts or the quality of the embryo transferred) than in our previous randomised control trial, when comparing LBR between the precision care and conventional care groups, in dysregulated patients. Collectively, these findings suggest a negative impact of endometrial immune dysregulation when this is not diagnosed, especially when the embryo is morphologically suboptimal and when some previous attempts have failed.

The use of mRNA ratios to precisely quantify some targeted immune expression in the endometrium is innovative. The normal range of expression had been accurately defined using a cohort of fertile patients. Noting that a balanced endometrial immune environment tends to perform better than a dysregulated one provides support for the clinical relevance of our interpretations.

Strong published evidence documented that the pregnancy results from a very early balanced immune dialogue between the embryo and the endometrium (19–21), indicating that an endometrial immune dysregulation may disturb this fundamental initial interaction. The endometrial immune environment, which has been historically overlooked clinical practice, is now considered as a critical factor enhancing ART performance (21–23). Accumulating clinical trials report the essential role of immune cells in endometrial receptivity for embryo implantation and early placental development (21, 23–25).

Based on extensive cohort studies (12–14, 24, 25) and a randomised control study (11), 78% of infertile patients are diagnosed with endometrial immune dysregulation. This marked percentage suggests that the endometrial immune profile may not necessarily indicate a pathological condition in the uterus, but rather represents a less balanced environment for implantation. It is noteworthy that 29% of patients experiencing dysregulation under conventional care successfully delivered after ET. The influence of the endometrial immune dysregulation seems to exert minimal effects in women who have never undergone ET or when embryos with optimal morphology have been transferred.

However, for most patients with previous implantation failures or morphologically suboptimal embryos (noted in 65% of the reported cases), an endometrial immune dysregulation may negatively affect LBR, while a balanced endometrial immune environment may be an advantage in young IVF patients.

Indeed, in contrast to the high LBR observed in this young IVF group with a balanced environment, large previous cohort studies including patients with a history of RIF revealed that patients with a balanced immune profile had a lower live birth rate (LBR) than those with treated dysregulation. One possible explanation is that, in patients with RIF, balanced immunity indicates embryo-related issues, whereas in patients with a good prognosis, it predicts success. In the early stages of development, it has been reported that the embryo possesses a significant capacity to overcome local endometrial immune dysregulation (26). As outlined in the latest research, an embryo has the capacity to promote adhesion prior to implantation (27, 28) and secrete immunosuppressors contained in extracellular vesicles (29, 30). However, the majority of embryos generated in cases of infertility may not be fully competent from an immune perspective, and local immune dysregulation may exert deleterious effects if left untreated. Chavli et al. (31) applied single-cell whole-genome sequencing to the trophectoderm and inner cell mass of 55 morphologically high-quality blastocysts. Mosaic karyotypic variation, encompassing both numerical and structural chromosomal changes, was detected in 82% of embryos (31). The rarity of overt chromosomal abnormalities in established pregnancies suggests that many of these early lesions are transient, being corrected, selectively eliminated, or both. Further studies are needed to determine whether a balanced immune environment contributes to these repair processes.

To improve overall outcomes, an integrated model including both the embryo and the endometrium is essential (32). Detecting an endometrial immune imbalance, especially when embryos are morphologically suboptimal, is crucial for improving subsequent outcomes. Endometrial immune profiling seems to be an effective approach for detecting such imbalances. Further studies should establish if rebalancing the uterine immune environment would be a solution to sustain the implantation of morphologically non optimal embryos.

## 5 Limitations

The data presented in this study should be interpreted with caution due to the limited number of transfers in the subgroup analysis, the multifactorial actors involved in the implantation process and due to the inherent structural limitations related to open-label human IVF trials. During the eight years of recruitment, there were several changes in IVF practice, such as a shift from transferring embryos at the cleavage stage to the blastocyst stage. To ensure that no deviation was observed during the study, a sensitivity analysis has been performed adjusted over calendar year. The single-centre design has its advantages and disadvantages: it minimises variability in practice over time, but also limits the generalisability of the results to other settings. In addition, replication of these results in patients with repeated implantation failure and older populations requires careful consideration of potential confounding factors, such as the ploidy of transferred embryos. In terms of uterine immune profiling itself, the lack of immune profiling for other key immune cells such as macrophages, dendritic cells, T regulatory cells is a limitation, as these cells are also known to be critical in the implantation process. Moreover, quantifying the individual impact of each of these factors is hard, and assessing their combined effect is even more complex.

## 6 Conclusion

Incorporating the “endometrial immune environment” as a variable in the dataset for managing infertile patients appears to be critical. A balanced endometrial immune environment appears as a positive indicator when using morphologically non-optimal embryos or with a history of at least one implantation failure. In contrast, no deleterious impact of uterine immune dysregulation was observed in patients using morphologically top-quality embryos, a low range of transfer suggesting that the embryo itself can regulate local dysregulation. Further controlled cohort studies and multicentric randomized trials are required to elucidate the differences observed regarding the uterine immune environment in relation to the embryo quality.

## Data Availability

The datasets generated and analyzed during the current study are available from the corresponding author upon reasonable request and subject to approval by the institutional review board.
